# Extra-Uterine Fœtus, Successfully Removed

**Published:** 1826-07

**Authors:** 


### EXTRA-UTERINE FŒTUS REMOVED WITH SUCCESS.

This formidable operation has been successfully performed, it appears, by a Mr. Bulh in Germany, during the last year. The woman was 36 years of age, and had borne children in the natural way. A tumour rose in the umbilical region, and at length broke in two places, discharging some pus. A surgeon enlarged one of the openings, when a quantity of sanies came away, together with portions of skin and hair. On farther examination it was ascertained that there was an extra-uterine fœtation, and it was determined that gastrotomy should be performed. An incision was made, from above the umbilicus to within an inch or two of the pubes, when the fœtus presented itself, and was removed. The umbilical cord was traced to the side of the uterus, and there it was seen implanted into a soft vascular network, which was probably the placenta. The whole was removed, the fluids sponged up, and the wound brought together by means of sutures. Inflammatory symptoms came on next day, and rose to an alarming height; but these were subdued, and the poor woman completely recovered, so as to be able to leave her bed on the 55th day from the operation.—*Archives.*

